# Diurnal Variability in Chlorophyll-a, Carotenoids, CDOM and SO_4_^2−^ Intensity of Offshore Seawater Detected by an Underwater Fluorescence-Raman Spectral System

**DOI:** 10.3390/s16071082

**Published:** 2016-07-13

**Authors:** Jing Chen, Wangquan Ye, Jinjia Guo, Zhao Luo, Ying Li

**Affiliations:** Optics and Optoelectronics Laboratory, Ocean University of China, Qingdao 266100, China; chenjingcj40@163.com (J.C.); jxyewaqu@163.com (W.Y.); opticsc@ouc.edu.cn (J.G.); lo-qd@hotmail.com (Z.L.)

**Keywords:** fluorescence-Raman spectral, chlorophyll-a, carotenoids, chromophoric dissolved organic matter, sulfate, in situ detection

## Abstract

A newly developed integrated fluorescence-Raman spectral system (λ_ex_ = 532 nm) for detecting Chlorophyll-a (chl-a), Chromophoric Dissolved Organic Matter (CDOM), carotenoids and SO_4_^2−^ in situ was used to successfully investigate the diurnal variability of all above. Simultaneously using the integration of fluorescence spectroscopy and Raman spectroscopy techniques provided comprehensive marine information due to the complementarity between the different excitation mechanisms and different selection rules. The investigation took place in offshore seawater of the Yellow Sea (36°05′40′′ N, 120°31′32′′ E) in October 2014. To detect chl-a, CDOM, carotenoids and SO_4_^2−^, the fluorescence-Raman spectral system was deployed. It was found that troughs of chl-a and CDOM fluorescence signal intensity were observed during high tides, while the signal intensity showed high values with larger fluctuations during ebb-tide. Chl-a and carotenoids were influenced by solar radiation within a day cycle by different detection techniques, as well as displaying similar and synchronous tendency. CDOM fluorescence cause interference to the measurement of SO_4_^2−^. To avoid such interference, the backup Raman spectroscopy system with λ_ex_ = 785 nm was employed to detect SO_4_^2−^ concentration on the following day. The results demonstrated that the fluorescence-Raman spectral system has great potential in detection of chl-a, carotenoids, CDOM and SO_4_^2−^ in the ocean.

## 1. Introduction

Marine analytical chemistry plays a key role in the understanding of marine systems, as chemical cues constitute much of the language of life in the sea [1,2,3]. Chlorophylls and carotenoids play a crucial role in absorbing and transferring light energy for the photosynthetic processes of marine organisms [4], which are commonly used as quantitative biomarkers for monitoring abundance and the composition of phytoplankton [5,6]. As an important constituent in shaping aquatic ecosystems, CDOM strongly absorbs ultraviolet light, which affects living organisms [7,8]. As the second concentrated inorganic salt in the ocean, sulfate has an important impact on the global sulfur cycle and climate change [9,10]. Therefore, detecting these chemical parameters of interest simultaneously is a benefit of studying the regional ecological environment.

However, these chemical parameters of interest vary widely in time and space and cannot be characterized by infrequent fixed interval sampling [11,12]. Typically in marine chemistry, the samples are analyzed after being taken back to the laboratory [13,14]. The “collection-transportation-measurement” procedures are usually labor- and time-consuming. In addition, changes of chemical composition and characteristics may happen due to the change of some parameters of the ambient environment, i.e., temperature and pressure during such procedures, which hinders further improvement of the accuracy and precision of the measurement [15]. In situ instruments are necessary in order to improve the accuracy and increase spatial and temporal resolution of sampling [16,17]. Optical methods, especially fluorescence spectroscopy and Raman spectroscopy, are regarded as promising techniques for in situ detection in the ocean [18,19,20].

The application of laser fluorescence and Raman in situ spectrometers has been thriving rapidly in recent years. The in situ fluorescence as a proxy for distribution and concentration of terrigenous DOM were used by the University of Hamburg in Nordic Seas for the purpose of refining estimates of carbon exports into the Atlantic Ocean [21]. An underwater system with a fiber-optic fluorescence sensor is developed to measure the chl-a and CDOM concentrations at different depths in real-time mode in Russia [22]. The Raman system developed by Monterey Bay Aquarium Research Institute has already been used to analyze a variety of in situ geochemistry [23,24]. A fluorescence-Raman spectral system by Ocean University of China has developed and carried out several sea trials successfully.

To better understand the information acquired with the integrated system, it is critical to know more about optical characteristics of chl-a, CDOM, carotenoids and SO_4_^2−^. Excited by a laser of 532 nm, chl-a as a pigment of phytoplankton emits fluorescence, which is proportional to concentrations of chl-a [25]. Phytoplankton can be classified by measuring the fluorescence emission of chl-a around 685 nm [26,27]. Fluorescence is an easily measured property of CDOM and can provide full coverage of the CDOM emission spectrum (~300 to 700 nm) [28]. Hence, chlorophyll and CDOM fluorescence can be used to monitor plant response to the environment [29]. Measuring intensity of carotenoids with Raman spectroscopy is an effective method to quantify and analyze aquatic phytoplankton with high sensitivity [30]. Two strong characteristic bands of carotenoids can be observed in the Raman spectrum at 1100–1200 cm^−1^ and 1400–1600 cm^−1^ regions due to C–C(ν3) stretching mode and C=C(ν1) stretching mode, respectively [31,32]. The Raman peak of SO_4_^2−^ at 981 cm^−1^ can be prominently detected due to S–O symmetric stretching [33,34]. Therefore, Raman spectroscopy can be applied to the investigation of carotenoids and SO_4_^2−^. The comprehensive and simultaneous observation of the compositions above is feasible.

In this article, based on the optical characteristics of chl-a, CDOM, carotenoids and SO_4_^2−^, a fluorescence-Raman spectral system was employed to detect chemical compositions during 22–24 October 2014 offshore in the Yellow Sea. The purpose of the present study is to investigate the diurnal variation of chl-a, carotenoids, CDOM and SO_4_^2−^. The results have shown that the application of a fluorescence-Raman spectral system has promising prospects for in situ detection in the ocean.

## 2. Instruments and Experiments

### 2.1. Instrument Setup

In order to obtain comprehensive marine information and compare the quantitative data from the fluorescence-Raman spectral system and the backup Raman spectral system in the following experiments, the fluorescence-Raman spectral system with excitation at 532 nm and the backup Raman spectral system, with excitation at 785 nm sharing a single control system, are developed. However, limited by power supply, they were unable to work simultaneously.

**Figure 1:**
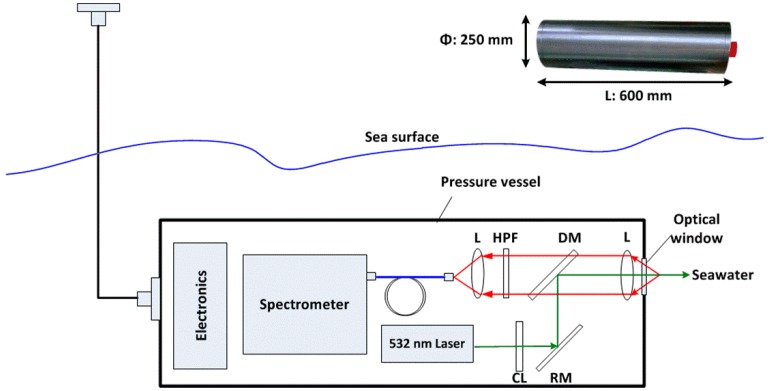
Schematic diagram of the fluorescence-Raman spectral system. (CL, collimator lens; RM, reflecting mirror; DM, dichroic mirror; L, focusing lens; HPF, high pass filter). The size of system is given at the upper right corner.

A fluorescence-Raman spectral system is used for in situ detection of Raman-active and fluorescence-active chemical constitutions in seawater. The system is 600 mm long and 250 mm in diameter. A schematic diagram of the various components is presented in Figure 1. A diode-pumped, solid state laser that emits at 532 nm and outputs power at 300 mW is used as the light source (LMX-532S, Oxxius, Lannion, France). The 532 nm excitation wavelength was selected because the efficiency of Raman scattering from a substance decreases as a function of λ^4^ and short wavelengths can excite greater fluorescence [35,36]. The spectrometer (QE65000, Ocean Optics, Dunedin, FL, USA) is compact and commercially available with a range of 532–700 nm (100–4500 cm^−1^). The wide spectral coverage is required so that the data can be collected in the low-wavelength range (for sulfate, carotenoids), mid-range (for H_2_O), and high range (for chl-a). The backscattering collection optical layout and the sapphire optical window are specially designed [37]. Particularly engineered, the fluorescence-Raman spectral system fits the requirement of marine monitoring.

The backup Raman spectral system has a structure similar to the fluorescence-Raman spectral system. A frequency-doubled laser operating at 785 nm and output power at 350 mW was chosen (I0785MU0350MF-NL, Innovative Photonic Solutions, Monmouth Junction, NJ, USA) because of its stability and high signal-to-noise ratio. The backscattering collection optical layout can accept optical signals with wavelength longers than 785 nm. The two systems employed spectrometers of the same type (QE65000). The backup Raman spectral system was set to collect Raman signals in the range of 300–2400 cm^−^^1^ (803–967 nm).

### 2.2. Experiment In Situ

**Figure 2:**
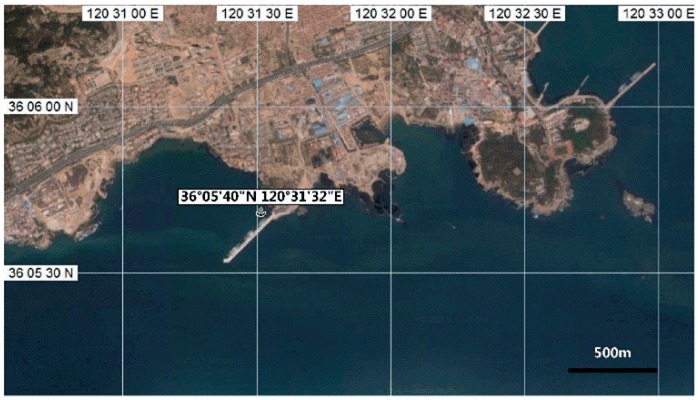
Map showing the location of in situ measurement in the Yellow Sea.

A series of shore-controlled experiments was performed offshore in the Yellow Sea by a fluorescence-Raman spectral system. The system was running during 14:00 22 October–8:14 23 October 2014. The sea trial was carried out on two sunny days immediately after three successional rainy days. Its temperature was reported from 13 °C to 19 °C and the experimental positions were exposed to level 3 to level 4 north wind on 22 October. The temperature was between 16 °C and 20 °C, with south wind scaled level 4 to 5 on 23 October. The transparency of seawater was 1.7 m by Secchi disc depth and the intensity of illumination was 19360 Lux by digital light meter at 03:05 p.m. 23 October. The observation station is 36°05′40′′ N, 120°31′32′′ E, which located on the continental shelf of the Yellow Sea near Shandong Peninsula (Figure 2). The site in which tides are regular semidiurnal tides is about 1.5 km from the nearest living district with a fishing port and 3.1 km from the nearest estuary of Han River. This area is of complexity in spatial and temporal distributions of hydrology, chemical constitutions and plankton under the influence by coastal current, the Yellow Sea cold water and Kuroshio, representing one of the important types of north temperate zones [38,39,40,41]. The rack-mounted system was deployed at a depth of 9 m on the continental shelf for the trial. Spectra were collected with intervals of 5 min and integrated time of 10 s.

### 2.3. Method of Data Processing

To acquire the information of spectra, spectral data were processed by a MATLAB compiler (MathWorks, Natick, MA, USA). This processing of fluorescence-Raman signals includes the following stages: finding of characteristic band position, noise removal, background correction and feature extraction. At the beginning, the continuous wavelet transform method was used to seek band position [42,43]. Then, the wavelet threshold method was applied to remove noise [44,45]. The fluorescence band of CDOM is much broader compared to the Raman band and the integral areas of CDOM were three or four orders of magnitude higher than the integral of sulfate, so the envelope of CDOM fluorescence spectrum in a wavelength range of 540–626 nm was approximated by exponential functions to obtain the features of CDOM before background correction. Penalized least squares method was employed to correct background later [46]. In the end, interested fingerprints could be extracted through curve fitting method and integration [47]. The features of characteristic bands including Al_2_O_3_, sulfate, carotenoids, H_2_O and chl-a were acquired from the original data.

The quantitative analysis of sulfate from the backup Raman spectral system was based on an internal standard normalization method. The intensity of a solute’s Raman signal in water can be described as R = IKPσC, in which R is the intensity of Raman signal, I is the excitation laser power, P is the effective optical path length, σ is the Raman cross-section of the object under investigation, and C is the concentration of object [48]. The water H–O–H bending mode near 1640 cm^−1^ and the band of Al_2_O_3_ (sapphire window) at 418 cm^−1^ are not sensitive to temperature or salinity. These two bands can be considered as constant, so they are considered as reliable concentration references. Since the information of water, Al_2_O_3_ and sulfate are measured at the same time, the multiple linear regression method described as $R_{SO_{4}^{2-}}^{*}=a(R_{SO_{4}^{2-}}/R_{Al_{2}O_{3}})+b(R_{SO_{4}^{2-}}/R_{H_{2}O})+c$ (a, b and c are constants) can compensate for the experimental fluctuation, such as laser power fluctuation and the instability of detection system. Therefore, based on the mentioned-above processing results, calibrated with this multiple linear regression method, the concentration of sulfate was retrieved.

For comparison with SO_4_^2−^ concentrations retrieved by in situ backup Raman spectral system, the samples were collected at 03:05 p.m. on 23 October. The samples were diluted by a factor of 500 with deionized water after the samples were carried back to the lab. Artificially prepared solution with SO_4_^2−^ concentration of 0.1, 0.2, 2.0, 4.0, 6.0, 8.0 and 10 mg/L, respectively, and diluted samples mentioned above were measured by a typical ion chromatography (Ics3000, Dionex, Sunnyvale, CA, USA) described in the research [49]. 

## 3. Results and Discussion

### 3.1. The Typical Spectra

**Figure 3:**
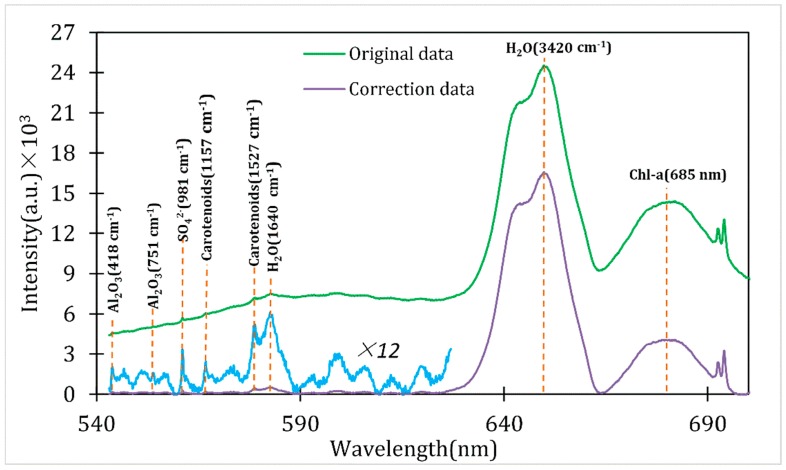
Typical spectrum acquired with and without correction by a fluorescence-Raman spectral system. The green line represents original data, obtained at 9:05 p.m. on 22 October, 2014. The violet line indicates the correction spectrum, and the blue line shows 12 times magnification of the correction spectrum within a range of 546.54–621.23 nm.

Typical results obtained with and without the above-mentioned background correction are shown in Figure 3. Two significant bands can be recognized to represent the fluorescence emission (around 685 nm) of chl-a in phytoplankton [50] and Raman emission (2750–3900 cm^−1^) of water H–O–H symmetric/anti-symmetric stretching modes [51] from the original data. CDOM fluorescence emission ranges from 300 to 700 nm. The excitation wavelength is 532 nm, and only emissions within the range of 532–700 nm can be detected. CDOM fluorescence range of 540–626 nm was selected for processing to avoid interference. To show the Raman spectra clearly, the signal was magnified 12 times after background correction. The bands of Al_2_O_3_ at 418 cm^−1^ and 751 cm^−1^ [52], S–O stretching of the sulfate anion at 981 cm^−1^ [53], water H–O–H bending mode at 1640 cm^−1^ [54], C–C symmetric stretching and C=C symmetric stretching [31] of carotenoids at 1157 cm^−1^ and 1527 cm^−1^ can be seen in Table 1. Among them, Al_2_O_3_ is the major component of sapphire window [55].

### 3.2. Diurnal Variation of Chl-a and CDOM

**Figure 4:**
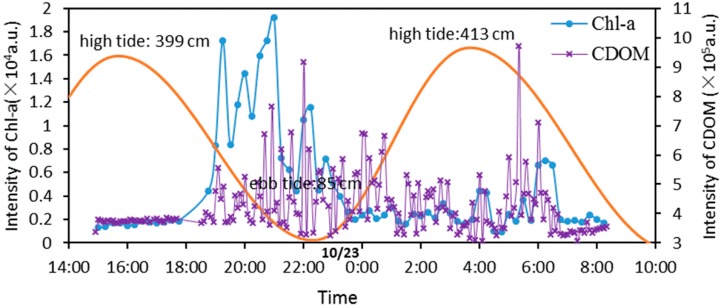
Diurnal variation of chl-a and CDOM fluorescence intensity. Blue symbols represent 15 min average intensity of chl-a, violet symbols indicate 5 min average intensity of CDOM. The orange line shows simulation of the tidal trend in the day, using tidal data from National Marine Data and Information Service as a reference.

Temporal change of chl-a and CDOM fluorescence signal are illustrated in Figure 4. To calculate the intensity of CDOM, the envelope of CDOM fluorescence spectrum in a wavelength range of 540–626 nm was approximated by exponential function [56]. Because chl-a fluorescence bands were separate in the spectrum, an integration from 672 nm to 695 nm using a Gaussian distribution after background correction processing was selected to acquire the intensity of chl-a at 685 nm [57]. Three spectra of measurements were averaged to obtain the characteristic information of chl-a.

It is found that chl-a signals remain constant during the daytime, while fluctuating drastically in the night. Chl-a are used as quantitative biomarkers of phytoplankton, which are the type that must be near the surface to survive and are similar to plants in that they must be in an environment in which photosynthesis can occur. Because the system was sitting on the seabed, chl-a signals were weak during the daytime and fluctuated drastically at night.

Troughs of fluorescence signal intensity of chl-a and CDOM were observed during high tides. By contrast, the signal intensity of chl-a and CDOM showed high values with larger fluctuations during ebb tides. The fluorescence signal intensity is positively correlated to the concentration. Certain correlations might exist between the tides and the concentration of chl-a and CDOM. One possible reason is that the phytoplankton may be dispersed with the high tides and may be enriched around ebb tides at the observation in which tides are regular semidiurnal tides. Tides play an important role in the aggregation and diffusion of algae. The tide could bring a lot of algae into coastal waters during high tides so that low chl-a signal intensity can be detected. With phytoplankton being enriched around ebb tides, deeper nutrient-rich water stimulated the growth and reproduction of phytoplankton so that high values of chl-a and CDOM with larger fluctuations can be detected by the system. According to the research, it is reported that two major peaks that represented the levels of chl-a in seawater at 1 m above the sea floor are obvious with values roughly twice those of the trough values of tidal current speed [58]. In addition, the tidal currents are tidal distance moved divided by the time elapsed. Therefore, the concentration of chl-a was affected by tides, which is in agreement with previous reports. However, the other reasons may be topography conditions of Yellow Sea, hydrological dynamics and nutrient of terrigenous input, which can contribute to intensity of different chl-a signals.

### 3.3. Diurnal Variation of Carotenoids

**Figure 5:**
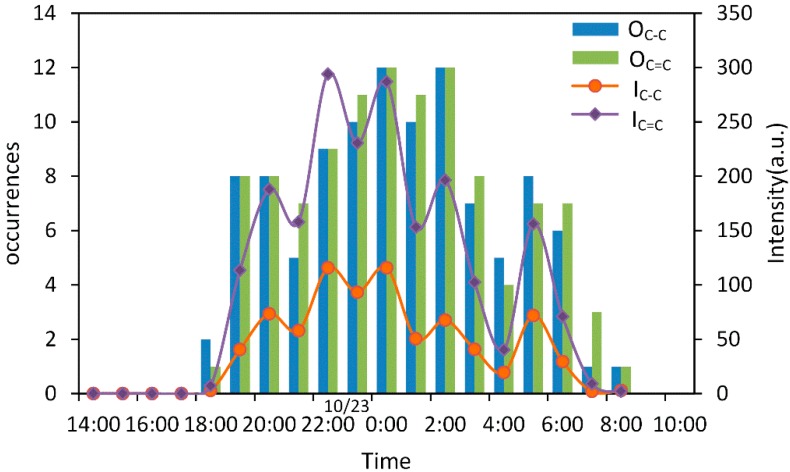
Hourly changes of occurrences and average intensity of carotenoid Raman signals resulted from C–C stretching and C=C stretching. (O_C–C_, the occurrences of C–C stretching mode; O_C=C_, the occurrences of C=C stretching mode; I_C_–_C_, the average intensity of C–C stretching mode of an hour; I_C=C_, the average intensity of C=C stretching mode of an hour). Blue and green histogram signify the occurrences detection of C–C stretching at 1157 cm^−1^ and C=C stretching mode of β-carotene at 1527 cm^−1^ (detection 12 times per hour), respectively. Orange lines and violet points represent average intensity of one hour of all above carotenoids.

In in situ experiments, signals of carotenoids can be detected or not detected with the change of the fluorescence background, so the signal was found to be discontinued. To analyze the diurnal variation of carotenoids, the occurrences (times of detection) per hour as well as the average intensity within each hour were counted. The results are demonstrated in Figure 5.

It can be seen that blue and green histograms signify the occurrences of C–C stretching at 1157 cm^−1^ and C=C stretching mode of α-carotene at 1527 cm^−1^ detected per hour, respectively. Orange and violet points represent average intensity within each hour. When the sun illuminated during 14:00–17:00 on 22 October, carotenoids were almost embedded in background noise. While in the night without solar radiation, the system can detect two bands with high Signal/Noise ratio, which reached a climax of intensity at 22:00. From 5:00 to 9:00 the next morning, the intensity of carotenoids declined gradually. It was indicated that carotenoids were influenced by solar radiation within a day cycle. By comparing Figure 4 with Figure 5, a consistency was found that the fluorescent signal of chl-a and the Raman signal of carotenoids were easily affected by solar radiation with different detection techniques, and also showed similar and synchronous tendencies. The reason may be that chl-a and carotenoids, which are both photosynthetic pigments on biomembranes of plankton, are commonly used as biomarkers for monitoring migration of plankton. Diel vertical migration is a widespread behavior in plankton [59]. Plankton are commonly used as biomarkers for monitoring migration of plankton. Diel vertical migration is a widespread behavior in plankton [60].

### 3.4. Temporal Change of CDOM and SO_4_^2−^

**Figure 6:**
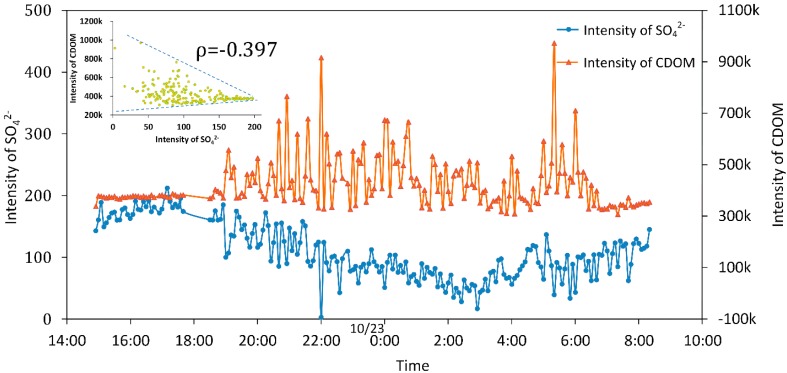
Temporal change intensity of SO_4_^2−^ Raman signal and CDOM fluorescence by a fluorescence-Raman spectral system. The figure at the upper left corner shows the significant negative linear relationship between SO_4_^2−^ Raman intensity and CDOM fluorescence with linear correlation coefficient of −0.397. Blue and orange points signify the intensity of SO_4_^2−^ and CDOM every 5 min, respectively.

Before this sea trial, we did some calibration experiments to establish a quantitative relationship between the SO_4_^2−^ Raman signal and sulfate concentration by a fluorescence-Raman spectral system with a series of artificially preparations whose true values of concentration were known. The information of SO_4_^2−^ concentration was expected. However, in the trial, the Raman signal of SO_4_^2−^ at 981 cm^−1^ (561.29 nm) was interfered with by fluorescence, especially CDOM emission spectrum ranges 300 to 700 nm. Figure 6 presents the relationship between intensity of SO_4_^2−^ and CDOM by the fluorescence-Raman spectral system. Blue and orange points signify the intensity of SO_4_^2−^ and CDOM every 5 min, respectively. A negative correlation was found between intensity of SO_4_^2−^ and CDOM with a linear coefficient of −0.397. 

**Figure 7:**
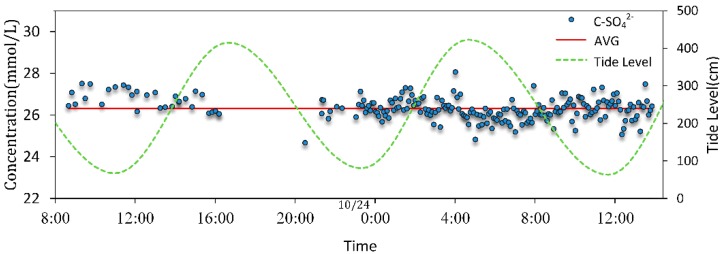
The temporal change of retrieved SO_4_^2−^concentrations detected by a Raman spectral system. (C-SO_4_^2−^, retrieved SO_4_^2−^ concentration; AVG, the average of SO_4_^2−^ during the day). The data between 16:12–20:30 were missing due to power off.

To avoid such interference, we employed the backup Raman Spectroscopy system (the excitation wavelength is 785 nm) to detect SO_4_^2−^ concentration from 8:14 on 23 October to 13:53 on 24 October. The Raman spectra of the Na_2_SO_4_ solutions with various concentrations (5, 10, 20, 30, 40, 50, 60 mmol/L) were acquired. Based on the above-mentioned processing results, with a multiple linear regression method, the concentrations of interest were predicted [61]. The temporal changes of retrieved SO_4_^2−^ concentrations detected by the Raman spectral system was shown in Figure 7.

It showed that the concentrations of SO_4_^2−^ ranged from 24.67 to 28.06 mmol/L with an relative standard deviation of 2.01% with average of 26.32 mmol/L, which was very close to that of measured by ion chromatography (Ics3000, Dionex, Sunnyvale, CA, USA) (25.69 mmol/L with 2.45% relative deviation). However, the SO_4_^2−^ concentrations measured by Raman spectral system and ion chromatography were at a lower of 28.25 mmol/L in standard seawater [62], and the cause might be sea water migration or terrigenous freshwater input. Through the published data, it was found that the salinity of seawater (usually 35‰) is made up of all the dissolved salts, and in this situation the concentration of chloride (Cl^−^), sodium (Na^+^) and sulfate (SO_4_^2−^) are 546 mmol/L, 468 mmol/L and 28.1 mmol/L, respectively [63]. However, the global salinity of the oceans changes slightly from around 32% to 40% (data from Woods Hole Oceanographic Institution). In the Yellow Sea, the salinity of surface seawater ranged from 30.2% to 31.0% [64] so that the concentration of sulfate is lower than 28.1 mmol. Furthermore, if the object was surface water of the coastal area, the salinity depended on the proportion of evaporation, precipitation, and terrigenous freshwater input. The sea trial was carried out on two sunny days immediately after three successive rainy days, and the experimental area, where tides are regular semidiurnal tides, is about 1.5 km from the nearest living district with a fishing port and 3.1 km from the nearest estuary of Han River. Through the published data, we found that the concentration of SO_4_^2−^ in the Xiangpu harbor in the East China Sea is 26.90 mmol/L (2582.34 mg/dm^3^ [65], which was very close to that measured by the backup Raman spectral system. Therefore, SO_4_^2−^ data retrieved during the experiments can be representative of the changes of SO_4_^2−^ in the environment. Unfortunately, however, the SO_4_^2−^ data of the fluorescence-Raman spectral system and the backup Raman spectral system were not collected synchronistically; thus, data at the same time could not be compared for quantitation.

## 4. Conclusions

CDOM and chl-a fluorescence spectra, and carotenoids and SO_4_^2−^ Raman spectra were observed in situ during 18 h using an underwater fluorescence-Raman Spectral System by the Ocean University of China. The results show that a certain correlation exists between the tides and the concentration of chl-a and CDOM. One possible reason is that the phytoplankton may be dispersed with the high tide while enriched around the ebb tide. Other reasons may be topographic conditions, hydrological dynamics or terrigenous input of nutrients. The results also show that chl-a and carotenoids were influenced by solar radiation within a day cycle with different detection mechanisms. The Raman signal of SO_4_^2−^ was interfered with by fluorescence. To avoid such interference, we also employed the Raman Spectral system with λ_ex_ = 785 nm to detect SO_4_^2−^ concentration on the following day. All of the obtained results demonstrated that the fluorescence-Raman spectral system has great potential in detection of chl-a, carotenoids, CDOM and SO_4_^2−^ in the ocean. In further work, the combination of a fluorescence-Raman spectral system and backup Raman spectral system will be applied for long-term observation of chemical compositions in the ocean.

## Figures and Tables

**Table 1:** Raman shifts and band location of the interesting components (λ_ex_ = 532 nm).

Interesting Components	Raman Shift (cm^−1^)	Band Location (nm)
Al_2_O_3_	418	544.10
	751	554.14
SO_4_^2−^	981	561.29
Carotenoids	1157	566.89
	1527	579.04
H_2_O	1640	582.85
	2750–3900	623.17–671.28
CDOM	**―**	546.54–621.23
Chl-a	**―**	685
